# Personality and Sport Experience of 20–29-Year-Old Polish Male Professional Athletes

**DOI:** 10.3389/fpsyg.2022.854804

**Published:** 2022-03-23

**Authors:** Paweł Piepiora, Zbigniew Piepiora, Justyna Bagińska

**Affiliations:** ^1^Faculty of Physical Education and Sports, Wroclaw University of Health and Sport Sciences, Wrocław, Poland; ^2^Faculty of Environmental Engineering and Geodesy, Wrocław University of Environmental and Life Sciences, Wrocław, Poland; ^3^Faculty of Economics and Management, University of Business in Wrocław, Wrocław, Poland

**Keywords:** sports psychology, personality, sport experience, physical activity, society

## Abstract

More and more research reports assert that sport experience has an influence on shaping the personality of athletes. This paper aims at validating the connection between personality and sport experience. The research subject of were young Polish male athletes (*N* = 1,260) aged 20–29, out of 42 sports disciplines, with sport experience ranging from 3 to 12 years. In order to test the personality of the research subjects, a five-factor model of personality called the Big Five was applied. Statistical calculations and analyses were carried out with ver. 25 of the IBM SPSS Statistics software. The research has shown that all personality traits correlate in a statistically significant way with sport experience (*p* < 0.001): there is a negative correlation of sports experience with neuroticism and a positive correlation with traits such as extraversion, openness to experience, agreeableness, and conscientiousness. Thus, we have identified a relationship between sport experience and personality traits of the researched Polish male athletes—the longer the sport experience, the lower level of neuroticism and higher levels of extraversion, openness to experience, agreeableness, and conscientiousness. Duration of sport experience has a considerable influence on forming the personality of Polish male athletes. However, the interpretation of just the results regarding high level of extraversion and conscientiousness distinguishes Polish male athletes (20–29 years old) from the Polish male population of non-training people.

## Introduction

Research on personality in sport is important to both the field of sports psychology and personality psychology, which aims at defining what causes various processes, predispositions, and mental functions to make a certain definite whole in a person, with coherent and integrated activities. These issues in the physical culture field are often raised upon description and explanation of various interindividual psychological and physical properties and behaviors of athletes or those who pursue physical culture goals, such as organizers, teachers, and educators (Jarvis, 1999). Personality in sport theoretical research background involves factor theories, sometimes referred to as trait theories, with the Big Five personality model frequently being used for this purpose. Because personality traits explain relatively constant human dispositions, their pursuit in repeated behavioral patterns is even more reasonable. The advantage of the Big Five model is that it explains some socially and culturally important behaviors which normally depend on several personality traits simultaneously. The Big Five is made up of five major traits or dimensions: neuroticism, extraversion, openness to experience, agreeableness, and conscientiousness, which can be classified disjointly. Neuroticism reflects emotional adjustment in relation to emotional imbalance, that is sensibility in terms of negative emotions, and includes six formally distinguished components: anxiety, angry hostility, depression, impulsiveness, vulnerability, and self-consciousness. Extraversion characterizes the quality and quantity of social interactions as well as activity level, energy, and ability to experience positive emotions and has six distinguished components: gregariousness, warmth, assertiveness, activity, excitement-seeking, and positive emotions. Openness to experience describes an individual’s tendency to seek and positively value life experiences, tolerance toward novelty, and cognitive curiosity and includes six components: fantasy, esthetics, feelings, actions, ideas, and values. Agreeableness describes a positive or negative attitude toward other people, an interpersonal orientation manifested in altruism in relation to antagonisms, experienced in feelings, thoughts, and actions. This dimension is comprised of trust, straightforwardness, altruism, compliance, modesty, and tendermindedness. Conscientiousness characterizes an individual’s degree of organization, persistence, and motivation in goal-oriented activities and describes a person’s attitude toward work. The components of conscientiousness are as: competence, order, dutifulness, achievement striving, self-discipline, and deliberation (McCrae and Costa, 2003).

It was assumed that the duration of sport practice has an impact on the athletes’ personality (Eysenck et al., 1982; Eyesenck, 1995). The longer the sport practice, the clearer outline of an athlete’s personality, and that is what distinguishes athletes from the general population (Piedmont et al., 1999; Paunonen, 2003; Watson and Pulford, 2004; Shrivastaval et al., 2010; Mirzaei et al., 2013; Steca et al., 2018). The shaping of an athlete’s personality alongside with gaining experience is associated with the acquisition of skills instrumental in dealing with stressful situations in sport (Piepiora, 2021a). This could be related to the specificity of sports rivalry and the psychological requirements which sport poses to athletes (Piepiora, 2021b). It was presumed that sports activity influences the personality and in turn the formed personality traits influence the undertaken decisions (Piepiora and Witkowski, 2020a). Upon taking up a sports task, athletes know their competences, know what they are able to do and assume that they can perform this task, regardless of the circumstances and difficulties posed by the opponent. And the differences in the value-to-behavior relations may result from normative pressures on the performance of specific personality behaviors (Piepiora and Witkowski, 2020b).

It is known from prior research of other nationalities that sport experience may have an impact on the personality development of athletes (Eysenck et al., 1982; Eyesenck, 1995; Piedmont et al., 1999; Paunonen, 2003; Watson and Pulford, 2004; Shrivastaval et al., 2010; Mirzaei et al., 2013; Steca et al., 2018). Therefore, we assume that the longer the sport experience, the more clearly the athletes’ personality is outlined, and that is what tells them apart from the general population. That is why it is worth examining how sport (training and competition) shapes the personality of Polish male athletes. Such relationships have not yet been verified in the aforementioned population (Piepiora and Witkowski, 2020a,b; Piepiora, 2021a,b; Piepiora and Piepiora, 2021). Accordingly, we have verified whether the obtained results in the Polish sports environment may confirm that sport is an example of a model personality moderator (according to the Big Five model: low neuroticism, high extraversion, openness to experience, agreeableness, and conscientiousness) and considered the use of sports activity as an important social element (Papacharisis et al., 2005). Taking the above into consideration, the aim of this paper is to verify the relationship between personality and sport experience of Polish male professional senior athletes: 20–29 years.

## Materials and Methods

### Participants

The research was carried out between 1 October 2015 and 30 September 2019, independently from the Olympic cycle. The data were collected throughout 4 years in order to include the maximum number of Polish athletes in the senior age from the widest possible array of sports disciplines. The subject of the study was 1,260 men, selected non-randomly and purposely from the population of Polish male athletes between the ages of 20 and 29. The inclusion criteria for the respondents were following: free will to participate, age range of 20–29 years, they had to represent at least the second sports class, several years of sports experience (3 years and up), a valid competition license, and documented sports achievements at different competition levels. The criterion of minimum 3 years experience was reasonable because these were the athletes who had already had sport results. The exclusion criterion for the study was the omission of any of the questionnaire items by the respondents.

The study population consisted of athletes “from the following sports disciplines: alpine skiing, American football, archery, athletics – long runs, athletics – short runs, ballroom dancing, basketball, beach volleyball, biathlon, bodybuilding, Brazilian jiu-jitsu, break dance, canoeing, cycling, equestrian, fitness, floorball, football, freestyle wrestling, futsal, handball, indoor volleyball, judo, ju-jitsu, karate kyokushin, kickboxing, mixed martial arts, mountaineering, Olympic karate, orienteering, Oyama karate, rugby, shidokan karate, shotokan karate, snowboarding, sport climbing, sport shooting, style taekwondo, swimming, tennis, tobogganing, ultimate frisbee” (Piepiora and Piepiora, 2021, p. 6297). The study was designed so that in each sports group, there were samples of 30 athletes from the same sport discipline. They included Polish medalists of the World Championship, European Championship, World Cup, European Cup, World Games 2017, and other prestigious international ranking tournaments. All the researched athletes train sport professionally (however, sport is not the only source of their income).

**Figure 1:**
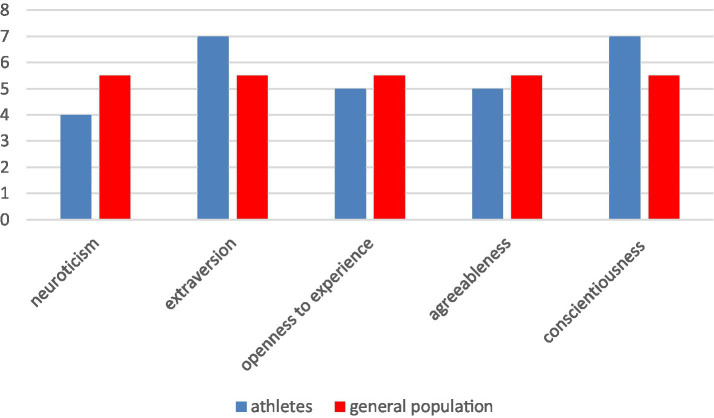
A column chart of the sten scores for personality profile of all tested athletes and the general population.

Mean values of personality traits of the studied Polish male population of 1,260 athletes aged between 20 and 29 were calculated, and their level was determined with the use of a sten scale. These results are summarized in Table 1. Then, the sten results of the entire studied Polish group of athletes and the mean of the general Polish population—which was 5.5 sten—according to the Polish version of the Personality Inventory NEO-FFI (that had been elaborated on the basis of a 2041-person sample) have been juxtaposed in Figure 1.

**Table 1:** Analysis of the mean and sten values of the studied athlete population according to personality traits.

**Variables**	All athletes (*N* = 1,260)	
	**M**	**Sten**
Neuroticism	14.4	4
Extraversion	31.26	7
Openness to experience	25.78	5
Agreeableness	28.1	5
Conscientiousness	34.47	7

The surveyed athletes were distinguished from the general population by a high level of extraversion and conscientiousness. It was found that in the dimensions of neuroticism, openness to experience, and agreeableness, the personality traits psychometrics of the studied athlete population did not differ from the general population.

### Research Tool

A five-factor personality model, known as the Big Five, was applied to examine the athletes’ personality with the use of the NEO—Five-Factor Inventory (NEO-FFI; Costa and McCrae, 2007). The NEO-FFI items are comprised of five measuring scales marked with abbreviations of the following factors: neuroticism, extraversion, openness to experience, agreeableness, and conscientiousness, forming a NEOAC acronym. The inventory comprises 60 self-report statements whose truthfulness was evaluated by the athletes on a five-point scale: “I definitely disagree”; “I disagree”; “I have no opinion”; “I agree”; and “I definitely agree.” The NEO-FFI has sten score norms for 5 age groups, established separately for males and females basing on big population samples. Additionally, the inventory is internally compatible. Its validity was proven by comparing the questionnaire results and the assessment of the respondents made by observers, as well as the correlation of the assessed traits with other dimensions of personality and temperament. Likewise, the factor validity was verified. The five-factor personality model meets the formal assumptions regarding personality traits. The Big Five dimensions are characterized not only by their universality and biological conditioning, but most of all by invariance and genuineness: they are a generalization of many personality characteristics and play an important role in the process of an individual’s adaptation to the environment. In this sense, NEO-FFI can be useful in the analysis of a number of psychological problems, both of theoretical and utilitarian nature. The results enable a comprehensive description of the respondents’ personalities within the Big Five framework and can be instrumental in forecasting their adaptation possibilities to the sport environment. Costa and McCrae (2007) assumed that in psychometrics, the results between 1 and 3 sten should be treated as low, and results from 7 to 10 sten—as high. The results ranging from 4 to 6 should be interpreted as average.

### Procedure

All the tested athletes consented to take part in the research after acquainting themselves with the information on its objectives and principles, likely effects, and potential benefits of the study. Moreover, the respondents understood the risk associated with taking part in the study, as well as its mode and the withdrawal possibility at any stage. The respondents could also ask questions and get answers. All tested athletes consented to the processing of data related to their participation in the research. The research was carried out in quiet rooms and the respondents were given 60 min to respond to the Inventory statements on paper. Moreover, the research was done in groups of maximum 30 people. After the completion of this research stage, the participants’ data were encoded.

The project received a positive opinion for using the research results issued by the Senate Committee on Ethics of Scientific Research at the Wroclaw University of Health and Sport Sciences, number 20/2019.

### Statistical Analysis

Statistical descriptive analyses, Pearson’s linear correlation coefficient, and linear regression models were performed using IBM SPSS Statistics, version 25. All linear regression models, except for neuroticism, were performed using the least squares method. For the neuroticism regression, the Weighted Least Squares method was used due to the broken assumption of the homoscedasticity of variance. In the performed models, the rule of thumb was also used for removing outliers above |3SD| for standardized residue values.

## Results

**Figure 2:**
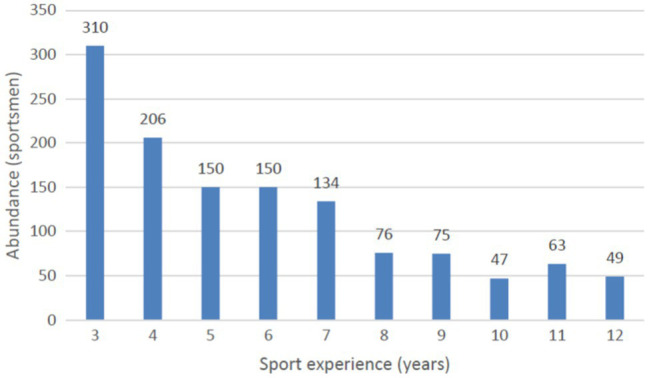
Distribution athlete groups regarding the sport experience measured in years.

In the first stage, the athletes were divided into 10 groups according to the length of sport experience measured in years, from 3 to 12 years (*M* = 5.85; *SD* = 2.67). Group sizes are shown in Figure 2.

The skewness value for the seniority measurement (Sk. = 0.76; Kurt. = −0.47) was within the adopted range; therefore, Pearson’s *r* correlation coefficients were used for the analyses.

It was found that all personality traits correlate significantly statistically with the sport experience of the surveyed athletes. A very strong (*r* > 0.7) and negative correlation was observed with the measurement of neuroticism. Weak and positive relationships (0.1 < *r* < 0.3) were noted for the measurements of extraversion, openness to experience, and conscientiousness. The relationship with agreeableness was very weak and positive (*r* < 0.1).

The following relationship between personality traits and sport experience among the surveyed Polish athletes was noticed as: the longer the sport experience, the lower neuroticism level, and the higher the remaining Big Five dimensions indicators. The results are summarized in Table 2.

**Table 2:** Coefficients of Pearson’s *r* correlation analyses between personality traits and sport experience among the surveyed athletes.

**Personality traits**		**Sport experience**
Neuroticism	Pearson’s *r*	−0.82
	significance	<0.001
Extraversion	Pearson’s *r*	0.24
	significance	<0.001
Openness to experience	Pearson’s *r*	0.16
	significance	<0.001
Agreeableness	Pearson’s *r*	0.09
	significance	0.001
Conscientiousness	Pearson’s *r*	0.30
	significance	<0.001

In the last stage, a series of linear regression models was created for the relationship between personality traits from the Big Five model and the sports experience of Polish athletes measured in years. All the performed regression models turned out to be statistically significant, which means that for each personality trait, seniority was their significant predictor. Such results are partly the result of a very large research sample (*N* = 1,260); therefore, an important aspect when interpreting the results is the analysis of the effect size measured with the standardized Beta—ranging from −1 to 1.

The prediction power for the model with the neuroticism measurement turned out to be very strong and the largest among all the models made. The direction of the relationship between neuroticism and sports training was negative, which means that the longer the training period, the lower the level of neuroticism. The relationships between extraversion, openness to experience, and agreeableness, and experience in sports were, respectively, weak and moderately strong. However, their direction was positive, which means that the longer the period of experience in a given sport, the higher the level of extraversion, openness to experience, and agreeableness. The last relationship between conscientiousness and sports experience should be described as very weak, and its direction was also positive. This means that the longer the training period, the higher the level of conscientiousness. The results are presented in Table 3.

**Table 3:** Coefficients of linear regression models predicting individual personality traits based on sports experience in years.

**Personality trait**		***B***	***SE***	***Beta***	**|*t*|**	***p***	***R*** ^2^	***F***	***df***
Neuroticism^a^	(constant)	24.49	0.19		128.00	<0.001	0.84	6578.02^*^	1; 1,243
	experience (years)	−1.74	0.02	−0.92	81.11	<0.001			
Extraversion	(constant)	28.13	0.37		75.57	<0.001	0.07	88.72^*^	1; 1,251
	experience (years)	0.55	0.06	0.26	9.42	<0.001			
Openness to experience	(constant)	23.68	0.39		60.04	<0.001	0.03	35.33^*^	1; 1,256
	experience (years)	0.37	0.06	0.17	5.94	<0.001			
Agreeableness	(constant)	27.02	0.41		66.52	<0.001	0.01	8.94^*^	1; 1,253
	experience (years)	0.19	0.06	0.08	2.99	0.003			
Conscientiousness	(constant)	30.30	0.40		76.01	<0.001	0.10	142.67^*^	1; 1,249
	experience (years)	0.74	0.06	0.32	11.94	<0.001			

* *p* < 0.001.

a With WLS method.

## Discussion

The aim of this paper is to verify the relationship between personality and sport experience of Polish male professional senior athletes aged 20–29 years. It has been shown that all personality traits correlate significantly statistically with sport experience: negatively with neuroticism and positively with extraversion, openness to experience, agreeableness, and conscientiousness. It was found that there is a relationship between personality traits and sport experience among the surveyed Polish male athletes between 20 and 29 years of age—the greater the sport experience, the lower the level of neuroticism, and higher markers of extraversion, openness to experience, agreeableness, and conscientiousness. This statistically significant result is probably a consequence of a large research sample and meets the research goal. The relationship between personality and sport experience in the Polish sporting environment has been confirmed. The longer the competition period, the clearer the personality of the athletes. The development of an athlete’s personality, alongside with gaining experience, is connected with the acquisition of the ability to cope with stressful situations in sport. This result fully applies to the dispositional theory of Zimbardo et al. (2012) stating that temperament is “the foundation of personality, deeply rooted in our individual biological nature” (Zimbardo et al., 2012, p. 422). Traits are treated as multiple dimensions of personality, characterizing to some extent the personality of each person. Traits are responsible for the consistency of personality in different situations and can be conditioned both by heredity and by learning. Bearing the above in mind, it was confirmed that the frequency of undertaking sports competition positively modifies human personality. It has been confirmed that sport shapes the personality of athletes (Eysenck et al., 1982; Eyesenck, 1995; Piepiora, 2019).

As we do not have prior knowledge about the personality of the examined athletes from the earlier periods of their sports careers, we have no foundation for determining how many years of sports training may have an impact on alterations of this vital human characteristic. Additionally, we do not know to what extent the specificity of trained sports and coaching might have had an impact on shaping the athletes’ personality (Harvey et al., 2020; Wallhead et al., 2021). The coach-athlete system is essentially a conflict system and it may be a desirable situation, as conflict often fosters development, and its absence may cause stagnation (Farias et al., 2015). Yet, the social and cultural factors cannot be excluded (Seippel, 2018). Therefore, the results of Polish athletes were compared with the Polish non-training population on the sten scale. It turned out that the Polish male athletes are distinguished from non-training Poles only by a high level of extraversion and conscientiousness. The other dimensions of personality are at an average level. This result highlights the importance of interpersonal relationships between athletes (high extraversion) and the impact of the training regime (high conscientiousness).

Our earlier study demonstrated differences between champions (Polish athletes with international sports successes) and other athletes (only with national, Polish, and sports successes) in all dimensions of personality in the Big Five model. The champions were distinguished by a “lower level of neuroticism and a higher level of extraversion, openness to experience, agreeableness and conscientiousness in relation to other athletes. But only neuroticism was a significant personality determinant predicting the level of achievement of the studied athletes: the lower the level of neuroticism, the greater the probability of an athlete being classified as a champion” (Piepiora and Piepiora, 2021, p. 6297). Previous studies have shown differences in the intensity of individual personality traits in combat sports (Piepiora and Witkowski, 2020a), individual sports (Piepiora, 2021b), and team sports (Piepiora, 2021a). The results of the study proved that there are differences in personality traits between athletes depending on the practiced sports, that is athletes show specific personality profiles according to the practiced sport. In each studied sport discipline, a different intensity of personality dimensions was found, and a different personality dimension was dominant. It was assumed that personality traits play a somewhat different role in the sport activity of athletes from different sports. The variation in the intensity of personality factors should be linked with the specificity of sports competition in the examined sports disciplines. Differences between champions and other athletes have also been found in combat sports (Piepiora and Witkowski, 2020a), individual sports (Piepiora, 2021b), and team sports (Piepiora, 2021a). The results suggest that the personality determinants of athletes are specific to particular sport groups. In each sport group, there were differences between champions and athletes in the factors of neuroticism and extraversion. The other dimensions were different depending on the sport group. In contrast, only one difference was found upon comparison between champions of each sport group. Combat sports champions demonstrated a statistically significant lower level of neuroticism in comparison with individual sports champions. This was presumed to depend on the physiognomy of the sport. Furthermore, a utilitarian factor characteristic only for combat sports became apparent (Piepiora and Witkowski, 2020b).

Sports activity affects the personality of people who do sports. In the athletic performance context, personality traits influence long-term athletic success, interpersonal relationships, and mental states of athletes before, during, and after the competition (Eysenck et al., 1982; Eyesenck, 1995). In the health-related exercise context, personality traits influence leisure time management, strength, and mobility in old age, but also some unhealthy or addictive physical behavior. Sport fosters the development of character, consistency in behavior and persistence in achieving goals (Hastie et al., 2011). The influence of sport on personality is evident. Sports competition is mostly about overcoming and testing oneself, revealing one’s abilities, predispositions, and skills; it is also a chance to overcome one’s weaknesses. Therefore, sports rivalry teaches an athlete how to follow the rules adopted in a given sports field, but also the general principles: equal chances and respect for the opponent (Witkowski et al., 2016). Without this, many achievements in sport, culture, or science would not have taken place. Development usually takes place through the clash of incompatible and competing views and their justifications. Furthermore, competition unquestionably kills boredom, brings animation, joy, and excitement. It generates an environment where people can fulfill their need for achievement. Through competition, the attractiveness of success also increases (Brock et al., 2009).

Thus, it is worth promoting sports activity among children, adolescents, and adults (Corneliu et al., 2012). The future use of sport activity as an important social component should be considered (Piepiora et al., 2021b). Sport teaches people to live in society. It is conducive to the development of character, consistency in behavior, and persistence in pursuing the goal (Piepiora et al., 2018). Also, sports competition is an opportunity to overcome one’s weaknesses, reveal one’s abilities, skills and predispositions, and test oneself. Thus, sports rivalry teaches an athlete not only how to follow the rules of a given sport, but also other common rules: equal chances and respecting the opponent (Cynarski et al., 2021; Piepiora et al., 2021a). Deprived of it, we would not have had numerous achievements in sport, culture, or science. Development frequently takes place through the clash of competing or incompatible viewpoints and their explanations. Competition also kills boredom, brings animation, excitement, and joy (Kalina, 2012; Evans, 2020; Petre et al., 2021).

Darwinian approach to sport is fully justified here (De Block and Dewitte, 2009). It takes into account a deeply cultural character and thus overcomes the traditional dichotomies of nature culture in the sport sociology. Sport should be viewed as culturally developed signaling systems that perform a function similar to the biological rituals of courtship in animals. Therefore, social learning underpins many aspects of cultural control of sport. And sport has developed new cultural functions because cultural evolution itself has become important to people (Bairner, 2012; Piepiora et al., 2016; Kalina and Kondzior, 2019; Klimczak and Kalina, 2020). Therefore, the Grand Unified Theory of sports performance is important: it assumes that the patterns of coordination and control, which directly determine the effect of performance, emerge from the confluence of interacting organismal, environmental, and task constraints through the creation and self-organization of coordination structures (Glazier, 2017). This theory provides the scientific basis for the integration of the sub-disciplines of sport sciences with the programs of support for applied sciences in sport adopted by international federations that govern various sports disciplines. But, on the other hand, it should be noted that the individual differences of athletes, as manifested in their ability to train, are at least partly determined by the genetic component. MTHFR A1298C appears to be one of the many polymorphisms involved (Cięszczyk et al., 2016). Therefore, we cannot exclude that genetic determinants influencing the performance of athletes may have influenced the respondents’ answers.

### Research Limitations

This study only covers the Polish population of male professional athletes aged 20–29. The study is limited by nationality, age, gender, and sports disciplines. Despite a large sample, it was not possible to test athletes from all sports disciplines trained in Poland. Therefore, the obtained results cannot be interpreted as universal. In the future, the study should be expanded to include other male and female age groups, possibly the largest possible population of athletes from all sports and nationalities. Our results can be a good point of reference for similar research, however.

### Utilitarian Value

Proper preparation of an athlete for sports competition includes factors related to physical and mental preparation. Therefore, the obtained research results may have great application during sports selection, training, and sports competition. They may constitute the basis for the development of suitable practical directives, important in the sports training of high-class players. In the sports selection of high-class players between the ages of 20 and 29 for the national male representation, the first verification stage may be the distribution level of personality traits. Candidates meeting the criteria of a model personality may be the desired individuals at the stage of mental selection. Only in the second stage, physical criteria should be taken into account, i.e., somatic build as well as motor, technical, and tactical predispositions as well as the achievements of the athletes.

## Conclusion

There is a relationship between personality traits and sport experience among the surveyed Polish male athletes between 20 and 29 years of age—the greater the sport experience, the lower the level of neuroticism, and the higher the levels of extraversion, openness to experience, agreeableness, and conscientiousness. But the interpretation of the results just in a high level of extraversion and conscientiousness distinguishes Polish male senior age athletes (20–29 years) from the non-training Polish male population. Therefore, extraversion and conscientiousness should be granted a leading role in shaping the personality through sport.

## Data Availability Statement

The original contributions presented in the study are included in the article/supplementary material, further inquiries can be directed to the corresponding author.

## Ethics Statement

The studies involving human participants were reviewed and approved by Senate Committee on Ethics of Scientific Research at the Wroclaw University of Health and Sport Sciences, number 20/2019. The patients/participants provided their written informed consent to participate in this study.

## Author Contributions

PP conceptualized and designed the study, organized the database, performed the statistical analysis, and wrote sections of the manuscript. PP, ZP, and JB wrote the first draft of the manuscript. All authors contributed to manuscript revision, read, and approved the submitted version.

## Conflict of Interest

The authors declare that the research was conducted in the absence of any commercial or financial relationships that could be construed as a potential conflict of interest.

## Publisher’s Note

All claims expressed in this article are solely those of the authors and do not necessarily represent those of their affiliated organizations, or those of the publisher, the editors and the reviewers. Any product that may be evaluated in this article, or claim that may be made by its manufacturer, is not guaranteed or endorsed by the publisher.
